# Putting conservation gardening into practice

**DOI:** 10.1038/s41598-023-39432-8

**Published:** 2023-08-31

**Authors:** Marius Munschek, Reinhard Witt, Katrin Kaltofen, Josiane Segar, Christian Wirth, Alexandra Weigelt, Rolf A. Engelmann, Ingmar R. Staude

**Affiliations:** 1 Institute of Biology, Leipzig University, Leipzig, Germany; 2 Die Naturgartenplaner, Regensburg, Germany; 3 German Centre for Integrative Biodiversity Research (iDiv) Halle-Jena Leipzig, Leipzig, Germany; 4 Botanical Garden of the University of Leipzig, Leipzig, Germany

**Keywords:** Ecology, Restoration ecology, Plant sciences, Plant domestication

## Abstract

Conservation gardening (CG) represents a socio-ecological approach to address the decline of native plant species and transform the gardening industry into an innovative conservation tool. However, essential information regarding amenable plants, their ecological requirements for gardening, and commercial availability remains limited and not readily available. In this study, we present a workflow using Germany as a case study to bridge this knowledge gap. We synthesized the Red Lists of all 16 federal states in Germany, and text-mined a comprehensive platform for garden plants, as well as multiple German producers of native plants. To provide accessible information, we developed a user-friendly app (https://conservation-gardening.shinyapps.io/app-en/) that offers region-specific lists of CG plants, along with practical guidance for planting and purchasing. Our findings reveal that a median of 845 plant species are red-listed across federal states (ranging from 515 to 1123), with 41% of these species amenable to gardening (ranging from 29 to 53%), resulting in a total of 988 CG species. Notably, 66% of these species (650) are already available for purchase. Additionally, we observed that many CG plants exhibit drought tolerance and require less fertilizer on average, with implications for long-term urban planning and climate adaptation. Collaborating with gardening experts, we present a selection of purchasable CG balcony plants for each federal state, highlighting the feasibility of CG even for individuals without gardens. With a multitude of declining plants amenable to gardening and the vital role of gardens as refuges and green corridors, CG holds substantial potential to catalyze transformative change in bending the curve of biodiversity loss.

## Introduction

Globally, species extinction rates are estimated to be between 10 and 100 times higher than background rates, with 40% of all plant species threatened with extinction^1,2^. Within Europe, 7–9% of vascular diversity is globally threatened across their whole range^3^. Concurrently, we are facing a decrease in opportunities of natural experiences^4^, with people in urban settings experiencing low or no connection or affinity to nature^5^. This in turn has implications for biodiversity conservation, as engagement to nature is a key predictor for conservation practices and behaviors^6^. Concordantly, high-level efforts to bend the curve of biodiversity loss^7^ have seen limited success. Biodiversity continues to decline even in protected areas, although at a lower rate than elsewhere, and between 50 and 80% are not managed effectively for meeting their basic goals, such as preventing species loss^8^. Indeed, management is often key to conserving plant diversity, making it costly ($76 billion annually) for protected areas to achieve set targets^9^. Whilst protected areas are and will remain the most important asset for nature conservation, there have been increasing calls for complementary and more participatory forms of conservation. One such form is conservation gardening (CG)^10^. In a rapidly urbanizing world, urban and rural green spaces can provide vast opportunities to engage the public in biodiversity conservation and leverage powerful social and economic mechanisms to address the biodiversity crisis.

CG is the seeding and planting of declining native plants in public and private, urban and rural green spaces. These spaces can serve as important refuges that offer suitable microhabitats, protection from disturbances, and management of competitive plants^10,11^. They can also act as launchpads for species dispersal, employing primary abiotic and biotic, or secondary anthropogenic dispersal mechanisms (including direct transplanting) into natural or restored habitats^10,12^. This could furthermore enhance the ability of declining species to track their ecological niches in a changing landscape^13,14^. Growing demand for such species could furthermore create an economic market and generate private revenue streams for declining native plant production and conservation efforts^10^. Additionally, in light of alarming declines in insect populations, CG can promote insect biodiversity, especially for those insects that are specialized on declining plants and are co-declining^15–17^. Studies suggest the features of a garden may more strongly influence pollinator diversity than the surrounding landscape, highlighting the vital role gardens can play in biodiversity conservation^18–20^. While conventional gardens often display high local plant diversity, a limited number of species dominate most gardens^21^. By focusing on regionally declining plants, CG has the benefit that cumulatively across regions more species are gardened. Together, CG could be a pivotal tool to bridge the gap between scientific knowledge and action for biodiversity conservation while unlocking private capital for conservation. Despite such potential benefits, putting CG into practice is beset by several challenges, currently hampering widespread societal uptake.

Increasing public awareness about the biodiversity crisis is a crucial initial step in overcoming one of the most pressing hurdles in CG. Despite surveys indicating an upward trend in awareness regarding biodiversity loss and a willingness to take action^22,23^, there is a strong and continued preference for tidy gardens with ornamental plants^24^. This barrier may be mitigated by raising awareness about the immediacy of the biodiversity crisis^1^ and emphasizing that gardeners can play a role in mitigating the crisis without sacrificing the aesthetic beauty of their gardens^10,25,26^. Beyond raising awareness, studies have underscored the efficacy of providing practical knowledge as one of the most potent methods for promoting CG among previously unengaged participants^27^. Plant selection and husbandry in gardens are heavily influenced by availability^28^ and personal experience^24^, highlighting the importance of fostering the easy availability of CG species. This issue is currently hampered by native seed availability. Whilst there are examples of seed producers in numerous countries, e.g., the members of the European Native Seed Producers Association, globally the market remains small, and in many countries native species remain non-existent on commercial markets (e.g., ref.^29^), with important negative effects on restoration activities in general^30^. Whilst native seed production is a necessary component of efforts to restore biodiversity, there is little political and industry momentum towards upscaled certified seed production. Creating public demand and awareness, as well as highlighting production gaps for declining species may be one vital pathway to upscale commercial availability. Public demand via CG could be a catalyst for this.

In this study, we aim to address the paucity in available and easily accessible CG plant lists by developing a user-friendly R Shiny application. Our focus is on Germany due to good data availability and because CG has already gained momentum in recent years (e.g., https://naturgarten.org/, https://tausende-gaerten.de/). To foster regionally specific demands that reflect species distributions and promote a framework that fully harnesses current monitoring knowledge, we synthesize Red List data from all 16 German federal states with text-mined data from NaturaDB, a comprehensive garden plant platform that integrated a plethora of sources on gardening plants (https://www.naturadb.de/info/quellen/). Our main objectives are to establish lists of declining native plant species amenable to gardening for each federal state, assess their commercial availability, evaluate their resilience to climate change, and make this information easily accessible to users. Using this database, we estimate the potential of CG to help mitigate the threat status of plants in Germany, assuming it becomes a mainstream approach. Additionally, we collaborate with practitioners to compile a selection of purchasable CG plants for balconies in each federal state. It is important to note that our approach to identifying CG plants is not exhaustive, as we do not consider factors such as species population stability, slug-resistance, symbiotic dependencies, or weediness. Similarly, our study presents an optimistic outlook on the potential role of gardens in biodiversity conservation that needs to be further validated by explicit field tests. Here, we aim to draw attention to the benefits of participatory conservation action and present a first step in implementing CG. Future efforts can build upon our findings to continually enhance our understanding of planting declining native species in gardens.

## Methods

### Database

First, we synthesized Red Lists from all German federal states. We obtained the most recent Red List of vascular plants for each state (median publication year: 2012) from the respective websites of the federal authorities. The Red Lists of Bremen and Lower Saxony were already combined, so that there were 15 distinct Red Lists for the 16 German federal states. Red List data were in pdf format and we used manual digitization and—where possible—Tabula (https://tabula.technology/) to transfer lists from pdf to csv format. Postprocessing of data (species standardization, author removal) was done using R. CG focuses on declining plant species, so we included red-listed species in the following categories: 0 (Extinct or Lost), 1 (Critically Endangered), 2 (Endangered), 3 (Vulnerable), G (Endangered—Unknown Extent), R (Rare), V (Near Threatened). We included extinct species (where the native habitats are likely lost as well) for the following reasons. Firstly, CG can create refuges for species currently extinct in the wild (e.g., *Franklinia alatamaha;* Maunder et al., 1998). Secondly, even if the native habitat has vanished, suitable conditions may still exist elsewhere or be created through human activities in the form of neo-habitats^31^, in which even extinct species may be re-discovered (e.g., *Arctostaphylos franciscana*). CG may therefore also benefit extinct species. There was some heterogeneity across federal states in how species were classified, with some Red Lists containing more threat (sub-)categories than others. We converted such categories to one of the aforementioned corresponding categories (e.g., R* to R). 

Second, we text-mined NaturaDB (https://naturadb.de) to collate data on species amenability to gardening. NaturaDB is a comprehensive database for German garden plants and provides practical know-how on how to garden them. For each species in our database, we pasted a NaturaDB URL (e.g., https://naturadb.de/pflanzen/alchemilla-alpina/) and searched for entries. Given that the plant was listed, we text-mined the following information from the species profile page: Common name, plant family, light requirements, water requirements, nutrients requirements, pH preference, soil type, frost tolerance, flower colour, height, associated biodiversity (butterflies, bees, birds, mammals), suitable for green roofs or balconies (y/n). Note that often not all of these categories had entries. Plant species not listed on NaturaDB were categorized as “not amenable to CG”; this need not be true of course, and our estimates for amenability are thus likely conservative. While considering NaturaDB as a comprehensive list of garden species, we also used a list based on the practical experience of our co-authors^32^ to assess how many of the non-CG plants may in fact be amenable to gardening.

**Figure 1:**
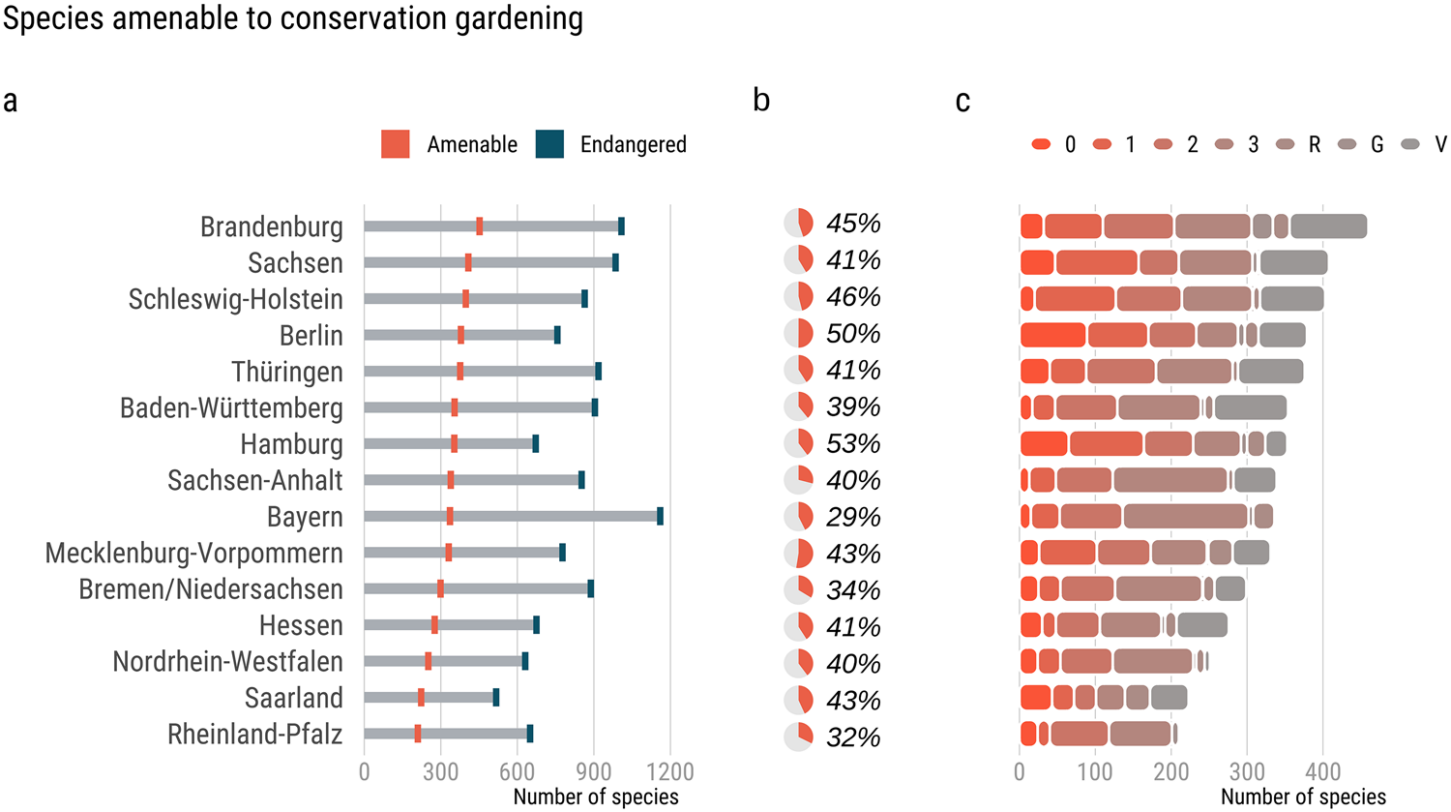
Between 29 and 53% of endangered species in German federal states are amenable to gardening, including extinct and critically endangered species. (**a**) Number of red-listed species listed per federal state (blue), and number of red-listed species also listed in NaturaDB aka Conservation Gardening (CG) species (orange). (**b**) Percentage of red-listed species that are amenable to CG. (**c**) Distribution of CG species across Red List categories (0 = Extinct or lost, 1 = Critically endangered, 2 = Endangered, 3 = Vulnerable, G = Endangered—unknown extent, R = Rare, V = Near threatened).

Third, we text-mined plant/seed producer websites to quantify the commercial availability of CG species. Our focus was on producers specializing in native seed and plant production, recommended by CG practitioners at Naturgarten eV (https://naturgarten-fachbetriebe.de/mitgliedsfirmen/wildpflanzen/). We text-mined databases from six producers: Gärtnerei Strickler (https://gaertnerei-strickler.de), Hof Berg-Garten (https://shop.hof-berggarten.de/), Rieger-Hofmann (https://www.rieger-hofmann.de/), Staudengärtnerei Spatz und Frank (https://staudenspatz.de/), Blauetikett Bornträger (https://blauetikett.de/), and Staudengärtnerei Gaißmeier (https://gaissmayer.de/). Although these producers are spread across Germany, we initially do not focus on provenance zones, as the phenology of plants along the urban–rural gradient differs from surrounding areas due to factors such as the urban heat island effect^33^, diminishing the role of local adaptations compared to restoration projects in open landscapes. However, local provenance, if available, is desirable. It is important to note that there are many more seed producers in Germany, and initiatives like "Tausende Gärten—Tausende Arten" aim to establish a Germany-wide network of plant producers to ensure sustainable production and genetic variability in native plants. From each producer, we gathered web shop URLs whenever a given CG species was commercially available. A given species may have multiple URLs if it was available for purchase from more than one producer. Moreover, a given species could have multiple URLs on one producer’s website if several varieties of that species were available for purchase. We included all URLs in our database. CG species that were not listed on any producer website were classified as “not produced”. Since we were not able to analyze the entire German market for native seeds and plants, it is possible that some plant species that we classified as “not produced” are actually available in Germany. See S Fig.1 for our workflow.

### Shiny app

**Figure 2:**
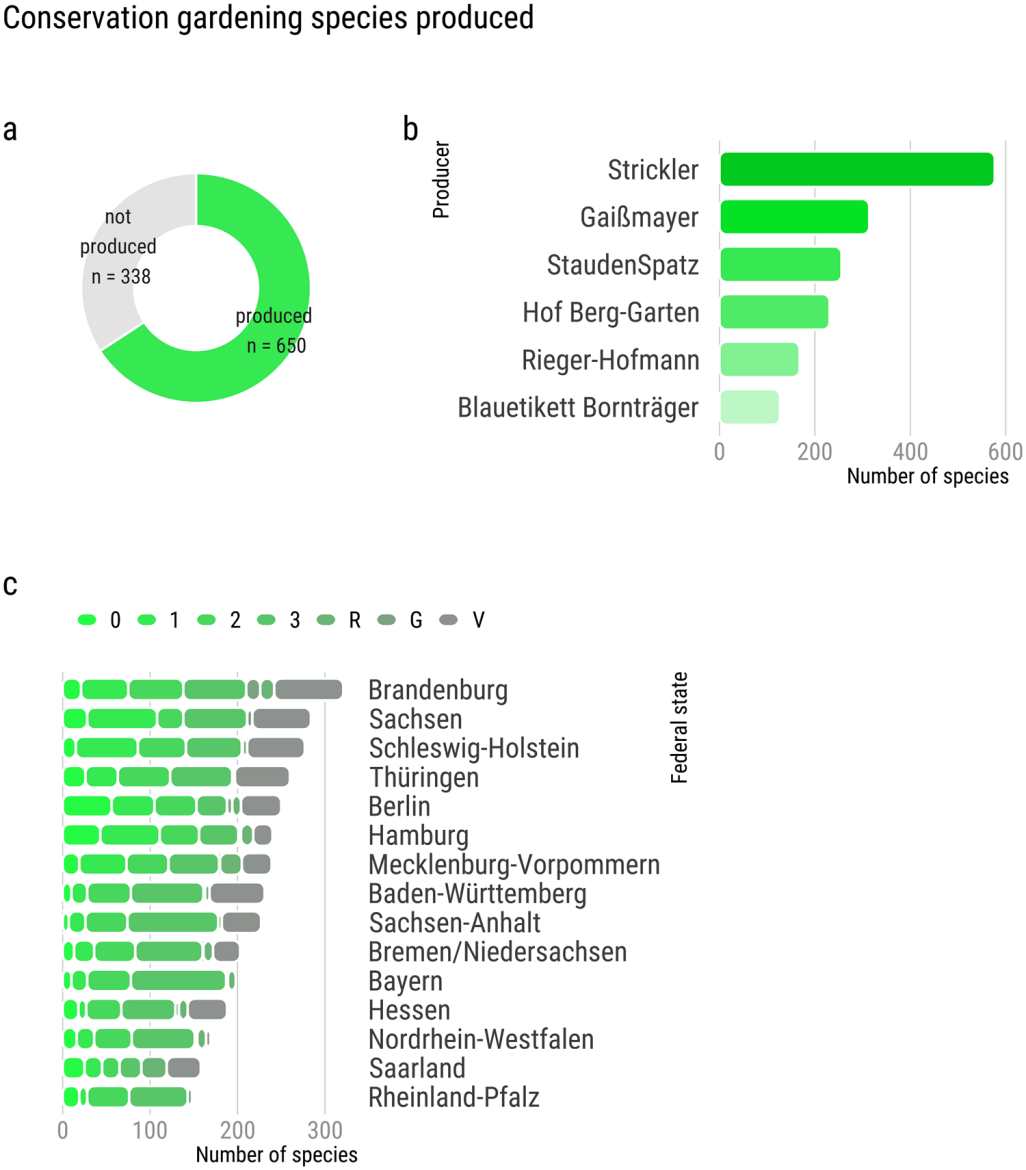
The majority (66%) of CG species is already produced and available for purchase. (**a**) Cumulatively across German federal states, we count a total of 988 distinct CG species. Of these, 650 species are already listed in the assortment of major native seed/plant producers in Germany. (**b**) Distribution of the number of produced CG species across producers. (**c**) Distribution of produced CG species in absolute numbers across Red List categories.

We created a user-friendly web application to provide comprehensive and easily accessible plant lists for gardeners and local authorities, facilitating informed plant selection (S Fig. 2). Users start by selecting their federal state, which filters the displayed CG species relevant to their region. Additional filters include horticulturally relevant categories (e.g., light demand) and species Red List category. Data downloads are available for each federal state for user convenience. Our application also features a “Producer” tab, enabling users to search for specific plants and obtain an overview of companies selling those species, including their online store URLs. The application also includes a “knowledge gap” tab, where users can download lists of plant species that we identified as “not amenable to CG” or “not produced”, thus highlighting knowledge and production gaps that could be closed in the future. Finally, the application includes a “Red List” tab, where we present an interactive map on which users can click on federal states to display all information pertaining to the underlying Red List. The web application was programmed and designed using the ‘Shiny’ package in R, and is available both in German (https://conservation-gardening.shinyapps.io/app-de/) and English (https://conservation-gardening.shinyapps.io/app-en/).

### Data analyses

NaturaDB does not list subspecies and varieties, therefore we were unable to take these into consideration. We only included taxa at the species level for all further analyses. 1) We quantified how many red-listed species are amenable to CG. We calculated the number of red-listed species per federal state. Next, we calculated the number and proportion of species amenable to CG per Red List category and per federal state. 2) We quantified the proportion of CG species available for purchase. We counted the distinct number of CG species across federal states, and quantified the total number and proportion of CG species available for purchase. We then proceeded to analyze these numbers for each producer and quantified how many purchasable species exist in each Red List category per federal state. 3) We contrasted CG plants with conventionally used garden plants in relation to drought resistance and fertilizer inputs. We obtained a list of the most commonly used garden plants in Germany from ref.^34^ (we presume that the identity of commonly cultivated plants has undergone minimal changes since the publication of the list 20 yrs ago). From this list, we included species in the following categories: Lawn and meadow plants, annual and biennial flowers, vegetables, garden and park shrubs, bulbous and tuberous plants, ornamental perennials and perennials. For these species, we queried water and nutrient requirements from NaturaDB, resulting in 134 conventionally used garden plants with water data and 307 with nutrient data. CG species counted 475 and 870 species with data on water and nutrient requirements, respectively. We quantified the proportion of species in each category for water and nutrient requirements, comparing CG species to conventionally used garden plants. 4) We estimated the potential of CG to reduce current threat levels for plants in Germany. We calculated current threat status in each federal state by dividing the number of red-listed taxa (including also varieties and subspecies) in the categories (0, 1, 2, 3, G, R, V) by the total number of taxa assessed in a given federal state (number provided in the respective Red Lists; no data on the number of assessed species were found for Rhineland-Palatinate). The reduced threat status with CG was calculated by subtracting the number of CG species (*n*_*cg*_) from the total number of red-listed taxa (*n*_*rl*_) and dividing by the total number of assessed taxa in a given federal state (*n*_*a*_): (*n*_*cg*_*–n*_*rl*_) */ n*_*a*_. We expanded this analysis beyond the level of federal states to the level of Germany as a whole. We used the German Red List^35^, identified CG species by text-mining NaturaDB, and again subtracted the number of CG species from the number of red-listed taxa in each Red List category to calculate the percentage by which CG could affect the threat status for vascular plants in Germany.

## Results

We found that the number of red-listed species (excluding subspecies and varieties) ranged from 515 to 1123 species across federal states, with a median of 41% being amenable to CG. In Hamburg, the proportion of red-listed species that were amenable to CG was highest (53%; 352 out of 670); in Bavaria, the count of red-listed species was highest (n = 1123), but only 29% of species were amenable to CG (n = 321) (Fig. 1a,b). A large portion of red-listed species amenable to gardening included highly endangered species, i.e., category 0, 1, 2, and 3 species. For example, in Berlin and Hamburg, 89 and 65 species listed as extinct or lost (category 0) are amenable to gardening according to NaturaDB (Fig. 1c). Cumulatively, across states 988 were classified as amenable to CG; 2474 species were classified as not amenable to CG, i.e., they were not listed in NaturaDB. We used an additional comprehensive list of 1000 native plant species amenable to gardening made by our co-authors^32^ to test the comprehensiveness of NaturaDB. We only found an additional 60 species, suggesting NaturaDB is a comprehensive source of gardening plants.

Of the 988 distinct CG species, the majority are already produced commercially (Fig. 2). 338 species were not yet found to be sold by six major native plant producers in Germany, highlighting a gap in potentially useful species for CG and a need for the development of more commercial regional plant nurseries. Yet, 650 species were already commercially available indicating that CG is in its infancy and that there exists a workforce and competency for timely upscaling (Fig. 2a). We found that one single producer “Strickler” produced the majority of these species (n = 577), with the other producers confining their assortment to a smaller subset (Fig. 2b). Notably, the produced CG species encompassed also the highest endangerment status. For instance, in Berlin, 56 species in the category 0 (extinct or lost) are available for purchase (Fig. 2c).

**Figure 3:**
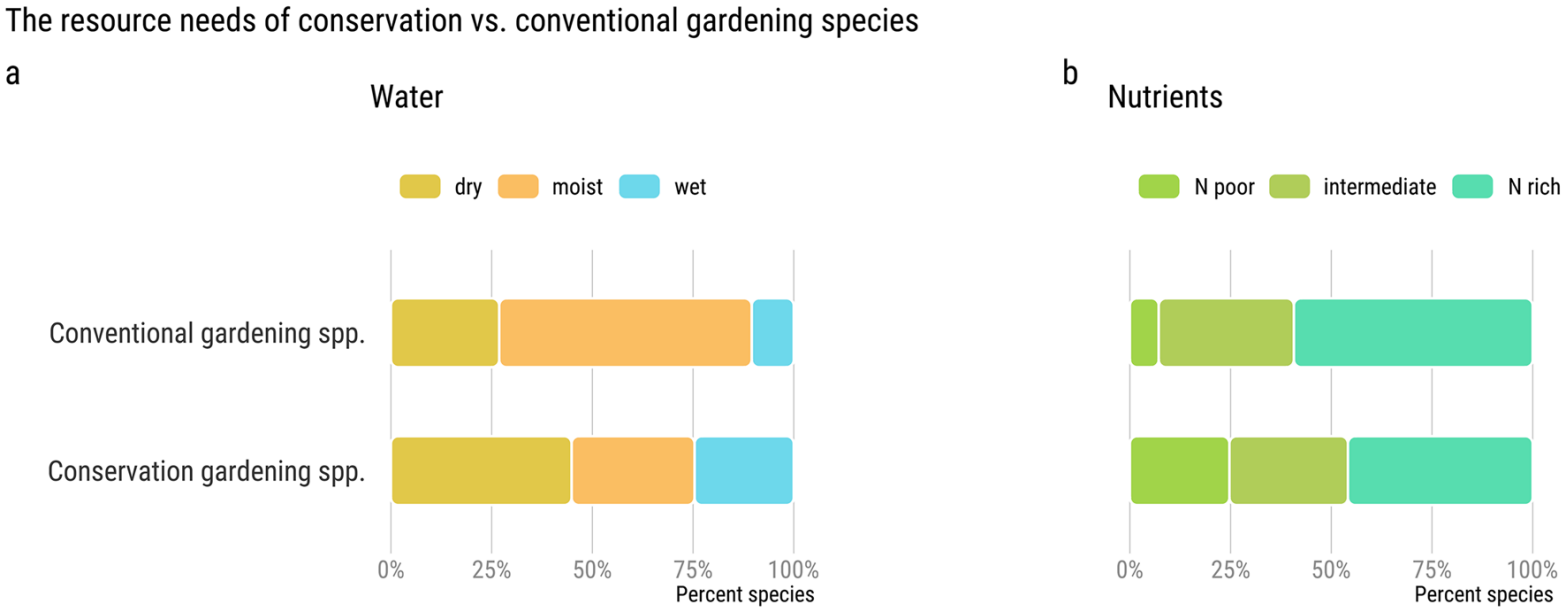
The average CG species is more likely to tolerate drought, and requires less fertilizer input. (**a**) Comparison amongst conventional gardening species (n = 134) and CG species (n = 475) in their water requirements (dry, moist, wet). (**b**) Comparison amongst conventional gardening species (n = 307) and CG species (n = 870) in their nutrient requirements (N poor, intermediate, N rich).

Against a backdrop of global warming with increasingly frequent and prolonged droughts, CG species appear to have an advantage over conventionally used garden species (Fig. 3). We estimate that 45% of CG species prefer dry soils compared to 27% of conventionally used garden plants (Fig. 3a). Interestingly, a high proportion of CG species also prefer wet habitats, such as riparian margins, where some of those species could be useful for wetland roofs as a measure to recycle grey water whilst mitigating the urban heat island effect^36,37^. CG species also require, on average, fewer nutrient inputs than the average conventional gardening species (Fig. 3b). 25% of CG species prefer nutrient-poor soils, as opposed to 7% of conventional gardening species, where the majority of species prefer nutrient-rich soils (60%).

**Figure 4:**
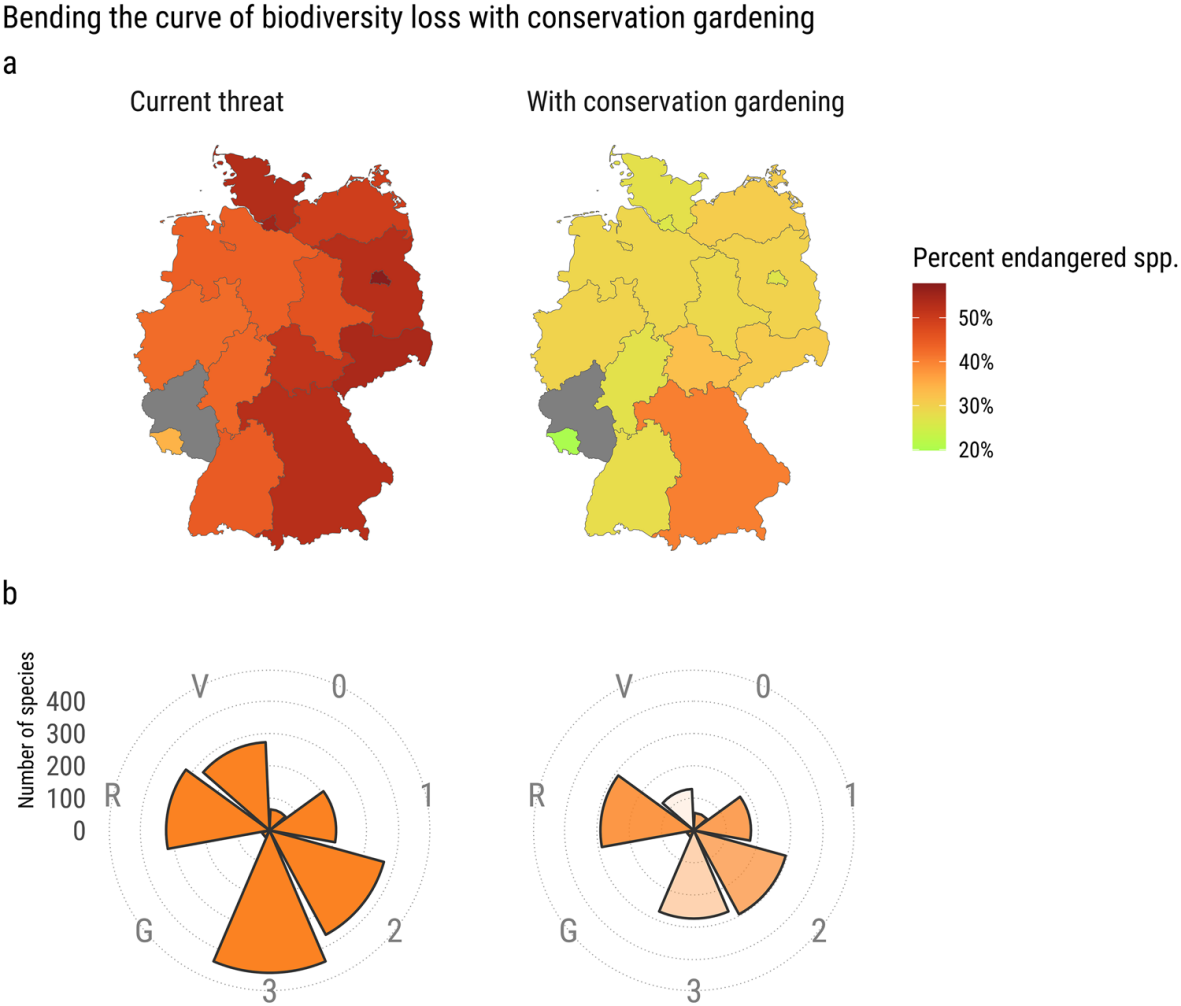
CG holds substantial potential to catalyze transformative change in bending the curve of biodiversity loss. (**a**) Current threat status versus potential threat status if CG were widely implemented (see Methods). (**b**) The transformative potential of CG at the level of Germany as a whole, using the 2018 Red List for the nation. Circular bar plots indicate the number of species in the Red List categories 0-V (left), and the number of red-listed species in these categories if CG would come to its full potential (right), i.e., subtracting CG species from the number of red-listed species. Maps were created in R, version 4.3.1 (https://r-project.org/).

CG has the potential to mitigate the decline of a substantial proportion of plants across the federal states of Germany (Fig. 4). Our estimates are based on the assumption that widespread implementation of CG could provide significant refugia and green corridors for threatened species, thereby mitigating their downward trajectory. Currently, the threat status, measured as the ratio of red-listed taxa to assessed taxa, ranges from 34% (Saarland) to 58% (Berlin) across German federal states, with a median of 50%. With CG, the median threat status could be reduced to 29% across states (Fig. 4a). The most substantial reductions could be achieved in Berlin and Hamburg, potentially reducing their threat status from 58 and 56%, respectively, to 26% each—a reduction of over 50% with CG. In Saarland, the threat status could fall as low as 19%. In Bavaria, where the number of threatened taxa is highest in Germany, the presence of subspecies and varieties, which we couldn't query (see Methods), partially explains the relatively higher threat level of 40% with CG (originally 53%), despite 321 species being amenable to gardening. While these numbers are specific to the state level, at the national level, using the 2018 German Red List, which currently red-lists 1689 taxa, we estimate that CG could reduce the threat status by 25%, with 460 species amenable to gardening (Fig. 4b).

**Figure 5:**
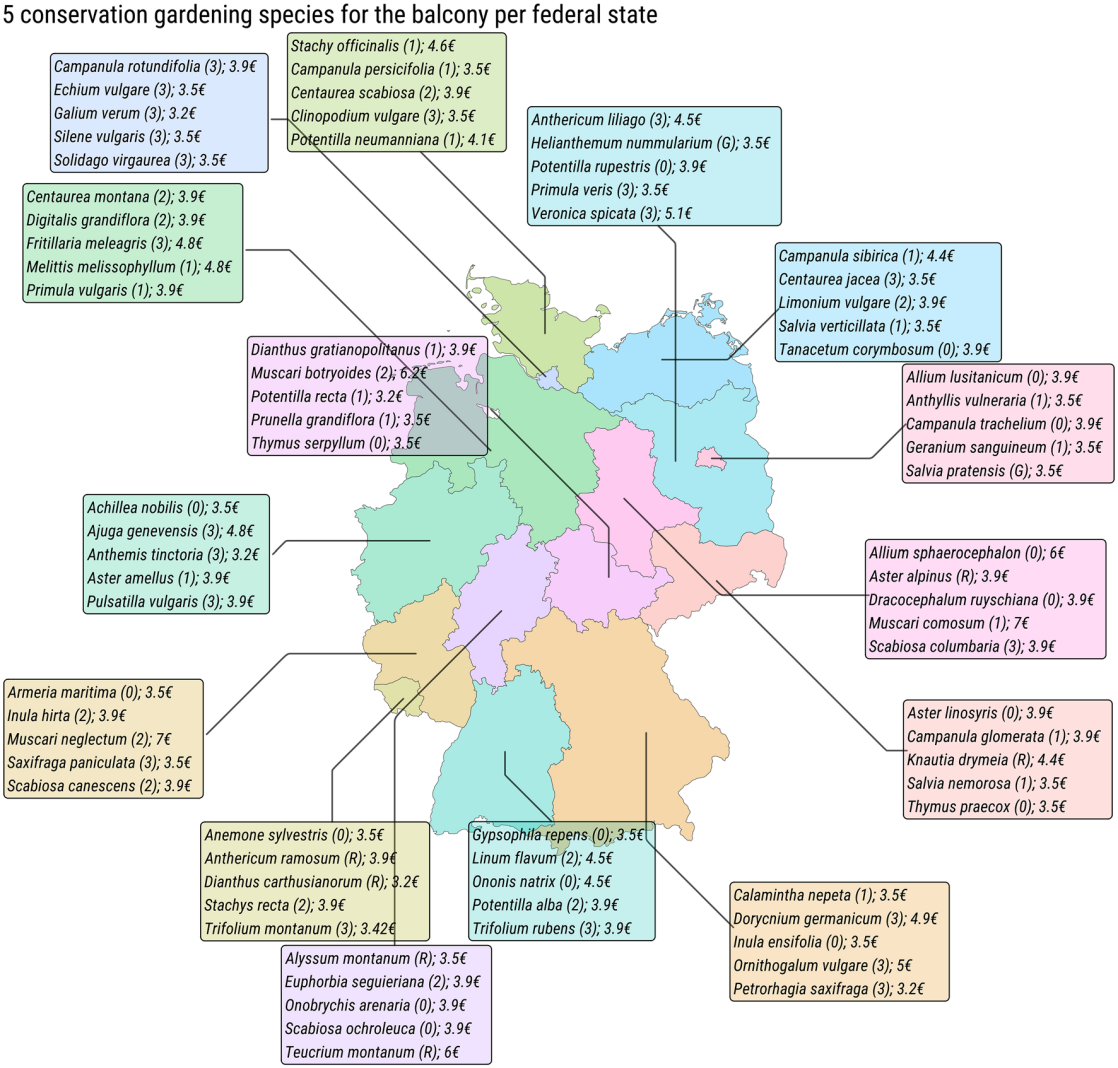
CG species for balconies for each federal state. Five exemplary CG species that can be grown on a balcony. Shown is the name of the species, the Red List category (in parentheses), and the purchase price (for clarity of availability, but note that prices are dynamic and apply here as of January 2023). For more planting possibilities for the balcony (also checked by practitioners) see Supplementary Table 2. Map was created in R, version 4.3.1 (https://r-project.org/).

To highlight some CG species that are already available for purchase, we provide five CG species that are suitable for being grown on a balcony for each federal state (Fig. 5). These recommendations are based on practitioner’s advice (from our co-authors) and on the data in our Shiny app, where species for green roofs or balconies can be filtered.

## Discussion

Based on our synthesis of German federal states' Red Lists and a comprehensive gardening platform, we estimate that on average 41% of red-listed species are potentially amenable to gardening. Many of these CG species were classified as highly endangered or even extinct. Despite this high threat level, the majority of CG species (66%) are already available for purchase by a few native seed producers. Beyond its potential to contribute to biodiversity conservation, we found that CG could also aid in climate adaptation of urban and rural green spaces. Compared to conventional garden plants, CG species more often prefer dry soils, indicating a greater tolerance to increasing droughts and lower water requirements that run in parallel with generally lower nutrient/fertilizer demands. Although further research is needed to fully understand the potential biodiversity benefits of CG, we offer an initial rough estimate. Large-scale planting of CG species in private and public green spaces, as an expanded and participatory form of ex situ conservation, has the potential to decrease the threat status of plants by up to 50% in certain German states and up to 25% across Germany as a whole. To fully harness this potential, it is critical for policymakers and the gardening industry to enhance the availability and promote the planting of regionally declining native species. In this process, the database we created and made accessible through an R Shiny application is one of many ways to both lower the threshold for gardeners to participate in CG and communicate key data and implementation gaps to policymakers and gardeners alike.

While we have argued that CG has significant potential to help address the biodiversity crisis, clearly more research is needed to substantiate this potential. In Germany, for example, 14% of the area is covered by settlements, and an even smaller fraction constitutes green spaces^38^. Relatively little area is thus available for CG to reverse biodiversity trends, which raises the question of how useful gardening can really be for biodiversity conservation. Although CG is not a panacea for native species conservation and many questions remain unanswered (see Outstanding Questions), we provide some reasons why CG could be a pivotal tool in addressing the biodiversity crisis. 1) Although urban green spaces may represent relatively little area, 70% of the German population lives in urban areas^39^, constituting a high human power for conservation purposes. Moreover, the human footprint (e.g., mobility infrastructure) extends far beyond settlements opening vast possibilities for secondary dispersal to natural ecosystems^40^. 2) Humans have been a key dispersal vector for plants throughout history and their importance has increased particularly recently^41,42^, with studies suggesting there is an especially high likelihood of human vectored dispersal in and away from urban areas (reviewed in ref.^43,44^). Despite the relatively small area, human infrastructure is widely dispersed in Germany. Even a square meter of human-managed vegetation could therefore create important habitat stepping stones^13,19^. 3) If there is an economic market for a particular species, i.e., if it is produced and there is demand, it is unlikely that the species will become extinct. 4) Beyond these direct contributions to mitigate biodiversity loss, CG has great educational value, making biodiversity accessible for city dwellers and raising awareness for its loss. This could in turn catalyze greater public demand for other conservation measures^6^.

Alongside these potentials contributions to biodiversity conservation, CG may also be associated with benefits for people, such as lowering costs and resources to maintain gardens. In Germany, the lion’s share of endangered plant species come from nutrient-poor and full-light conditions (e.g., dry calcareous grasslands)^45^. It is therefore to be expected that many CG species thrive under different site conditions than conventionally used garden plants. We found that CG species may be, on average, more tolerant to drought and water scarcity. In recent years, Germany has witnessed exceptional droughts^46^, making it costly and resource intensive to support so called “Wimbledon lawns”. Using CG plants instead could contribute to climate adaptation in rural and urban green spaces. Likewise, lower nutrient requirements of CG plants may reduce effort and costs for gardeners. Fertile soils are currently the norm amongst gardeners, with studies finding that 52% of gardeners apply fertilizers at least once a year^47^. We argue that the species that benefit from these activities are exactly those that are already winning in the Anthropocene^48^. Nutrient-demanding species are increasing inside and outside natural ecosystems alike. Clearly, it will also be important to assess the potential of CG plants to become weedy, i.e., overly-competitive and invasive. While the probability of weediness in declining native plants may be generally low, increasingly complete data on species invasiveness (e.g., https://glonaf.org/), will allow us to estimate this probability and plan accordingly. Taking such considerations into account, we suggest CG species could generally help reduce resource inputs and lead to more resilient garden ecosystems, whilst helping to safeguard biodiversity.

Our focus lies on declining native plants, but it's important to note that CG should not be limited to those species alone, as it does not assign a higher moral value to natives over non-natives. In fact, supporting a mixture of species can be desirable due to their complementary functional traits. Non-native plants, for instance, can provide resources for insects when native plants have finished flowering^49,50^. With the exception of gardens that plant invasives, most types of gardens already contribute to biodiversity, and CG should not be an exclusive approach to conservation. CG's focus on declining native plants is a systemic approach to enhance the cumulative diversity of plants across regions and put a spotlight on species at risk. A widespread shift towards predominantly native species in the horticultural industry could further help address Target 6 of the 2022 Kunming-Montreal Global Biodiversity Framework, which aims to mitigate the impacts of invasive species on biodiversity (https://cbd.int/article/cop15-final-text-kunming-montreal-gbf-221222). Garden escapes of non-native species often contribute to the spread of invasives^51^. By prioritizing declining native species, CG could eliminate a significant pathway for species invasions, and instead of being a costly intervention it might generate economic revenue. Lastly, it is crucial to recognize that many plants used in gardens are ornamental hybrids or varieties bred for aesthetics with low genetic diversity. Because CG can be similarly affected by such fashion trends, it is critical to establish industry standards that focus on the conservation of wild types, genetic diversity, and proximate provenance^10^. Leveraging established international native seed markets can greatly contribute to this effort.

Our app may help gardeners to gain an overview of the requisite knowledge for, and thereby lowering the entry threshold to CG. Nonetheless, our study and app have several shortcomings. Our calculations of how many plants are amenable to CG are currently not verified by practical experience, it only relies on the information provided by one comprehensive gardening platform. Important field trials are still lacking to determine which CG plants are easy to grow in gardens and which are not suitable for amateur gardeners. It would be useful to group plants in this way^10^. We take a step in this direction by providing recommendations for balcony plants (Fig. 5 and Supplementary Table 2), but a broader classification system for all CG plants is desirable. This expertise already, in part, exists among horticultural experts, however, it is often not made readily available for economic reasons. Programs that incentivize such knowledge sharing and mobilization could go a long way toward bridging the gap between science and practice. Furthermore, it is likely that our app still mainly reaches people who are already interested in natural gardening, and more research is needed on how to effectively lower the threshold to CG. For example, in allotment gardens, garden groups could take stewardship for a specific declining species, thereby creating communities that share seedlings, experience and knowledge of their “care plant” within the wider allotment community—a form of block leadership that may be effective in instigating community uptake^27,52^, and producers of seed mixtures could include at least 10% of regionally specific declining native plants. Here, botanical gardens would also be uniquely positioned to help promote CG and provide information on which plants are amenable to gardening.

Overall, we provide a workflow that leverages spatially detailed, subnational monitoring data, and integrates these data with gardening and producer platforms to find CG plants. Whilst we focus on Germany here, we hope that our workflow and software facilitate similar undertakings for other regions. We highlight that there is already considerable potential for the implementation of CG and propose purchasable CG plants for balcony gardening as a low-barrier entry point. Our application may not encompass the definitive list of CG plants and fall short regarding some elements of key practical advice, but we hope it will be informative to those who want to find a starting point and means to participate in tackling the biodiversity crisis.

Outstanding Questions

1. *Dispersal and establishment of conservation gardening (CG) plants:*
  o Do CG plants disperse from gardens and establish in natural/urban ecosystems?
    ▪ Conduct comparative studies between CG plant populations in gardens and adjacent natural/remnant habitats.
    ▪ Implement long-term monitoring and Before-After Control-Impact studies tracking the effect of CG on promoting dispersal and establishment beyond garden-boundaries.
    ▪ Determine the factors (e.g., proximity to source, habitat quality) that influence CG plant establishment.
    ▪ Explore dispersal and germination traits facilitating CG plant establishment.
    ▪ Study population dynamics and reproductive success of declining native plants in urban environments.
2. *Societal participation and CG success:*
  o How much societal participation is needed for CG to be successful as a conservation tool?
    ▪ Assess societal engagement in allotment gardens and its correlation with CG plant occurrence in the surrounding area.
    ▪ Implement experimental designs that manipulate the degree of societal participation in CG (e.g., residential blocks with a gradient of the number of balconies featuring CG plants) and compare CG plant establishment.
3. *Role of genetic diversity in CG:*
  o What is the role of genetic diversity of the plant material for CG to sustain viable populations?
    ▪ Compare survival and reproduction success of plant material with local vs. non-local provenance in urban gardens.
    ▪ Compare and track genetic structure of CG plants in gardens and spatially proximate natural habitats over time.

### Supplementary Information

Supplementary Information.

## Data Availability

R code for all data retrieval, carpentry, analyses and visualisations, as well as for the Shiny app is available under: https://github.com/istaude/conservation-gardening-shiny-app.

## References

[1] IPBES. Global assessment report on biodiversity and ecosystem services of the Intergovernmental Science-Policy Platform on Biodiversity and Ecosystem Services. *Bonn, Germany* (2019).

[2] Lughadha EN (2020). Extinction risk and threats to plants and fungi. Plants People Planet.

[3] Holz H, Segar J, Valdez J, Staude IR (2022). Assessing extinction risk across the geographic ranges of plant species in Europe. Plants People Planet.

[4] Cazalis V, Loreau M, Barragan-Jason G (2022). A global synthesis of trends in human experience of nature. Front. Ecol. Environ..

[5] Soga M, Gaston KJ (2022). Towards a unified understanding of human–nature interactions. Nat. Sustain..

[6] Soga M, Gaston KJ (2016). Extinction of experience: The loss of human–nature interactions. Front. Ecol. Environ..

[7] Mace GM (2018). Aiming higher to bend the curve of biodiversity loss. Nat. Sustain..

[8] Watson JEM, Dudley N, Segan DB, Hockings M (2014). The performance and potential of protected areas. Nature.

[9] McCarthy DP (2012). Financial costs of meeting global biodiversity conservation targets: Current spending and unmet needs. Science.

[10] Segar J (2022). Urban conservation gardening in the decade of restoration. Nat. Sustain..

[11] Ismail SA, Pouteau R, van Kleunen M, Maurel N, Kueffer C (2021). Horticultural plant use as a so-far neglected pillar of ex situ conservation. Conserv. Lett..

[12] Maunder M, Higgens S, Culham A (1998). Neither common nor garden: the garden as a refuge for threatened plant species. Curtis’s Botanical Mag..

[13] Rudd H, Vala J, Schaefer V (2002). Importance of backyard habitat in a comprehensive biodiversity conservation strategy: A connectivity analysis of urban green spaces. Restor. Ecol..

[14] Kirchner F, Ferdy J, Andalo C, Colas B, Moret J (2003). Role of corridors in plant dispersal: An example with the endangered *Ranunculus nodif lorus*. Conserv. Biol..

[15] Berthon K, Thomas F, Bekessy S (2021). The role of ‘nativeness’ in urban greening to support animal biodiversity. Landsc. Urban Plan.

[16] Griffiths-Lee J, Nicholls E, Goulson D (2022). Sown mini-meadows increase pollinator diversity in gardens. J. Insect. Conserv..

[17] Biesmeijer JC (2006). Parallel declines in pollinators and insect-pollinated plants in Britain and the Netherlands. Science.

[18] Majewska AA, Altizer S (2020). Planting gardens to support insect pollinators. Conserv. Biol..

[19] Witt, R. Natur für jeden Garten. 10 Schritte zum Natur-Erlebnis-Garten. In *Planung, Pflanzen, Tiere, Menschen, Pflege* (2013).

[20] Baldock KCR (2019). A systems approach reveals urban pollinator hotspots and conservation opportunities. Nat. Ecol. Evol..

[21] Pearse WD (2018). Homogenization of plant diversity, composition, and structure in North American urban yards. Ecosphere.

[22] Bundesamt für Naturschutz (BfN). *Nature Awareness Study*. (2019).

[23] Millard JW, Gregory RD, Jones KE, Freeman R (2021). The species awareness index as a conservation culturomics metric for public biodiversity awareness. Conserv. Biol..

[24] Goddard MA, Dougill AJ, Benton TG (2013). Why garden for wildlife? Social and ecological drivers, motivations and barriers for biodiversity management in residential landscapes. Ecol. Econ..

[25] Ignatieva M, Ahrné K (2013). Biodiverse green infrastructure for the twenty-first century: From “green desert” of lawns to biophilic cities. J. Archit. Urban..

[26] Lindemann-Matthies P, Marty T (2013). Does ecological gardening increase species richness and aesthetic quality of a garden?. Biol. Conserv..

[27] Shaw AE, Miller KK (2016). Preaching to the converted? Designing wildlife gardening programs to engage the unengaged. Appl. Environ. Educ. Commun..

[28] Smith RM, Thompson K, Hodgson JG, Warren PH, Gaston KJ (2006). Urban domestic gardens (IX): Composition and richness of the vascular plant flora, and implications for native biodiversity. Biol. Conserv..

[29] Rolim RG, Rosenfield MF, Overbeck GE (2022). Are we ready to restore South Brazilian grasslands? Plant material and legal requirements for restoration and plant production. Acta Bot. Bras..

[30] Ladouceur E (2018). Native seed supply and the restoration species pool. Conserv. Lett..

[31] Huhta A, Rautio P (2007). A case with blue gentian blues: Roadside-cutters creating neo grasslands as refugia for endangered *Gentianella campestris*. Nord. J. Bot..

[32] Witt, R. Wildpflanzen für jeden Garten: 1000 heimische Blumen, Stauden und Sträucher; Anzucht, Pflanzung. In *Pflege.-München, Wien, Zürich: BLV* (1994).

[33] Wohlfahrt G, Tomelleri E, Hammerle A (2019). The urban imprint on plant phenology. Nat. Ecol. Evol..

[34] Preisinger, H. *et al. Berichte des Botanischen Vereins zu Hamburg*. (2000).

[35] Metzing, D., Hofbauer, N., Ludwig, G. & Matzke-Hajek, G. *Rote Liste gefährdeter Tiere, Pflanzen und Pilze Deutschlands: Pflanzen/Redaktion: Detlev Metzing, Natalie Hofbauer, Gerhard Ludwig und Günter Matzke-Hajek*. (Bundesamt für Naturschutz, 2018).

[36] Zehnsdorf A (2019). Wetland roofs as an attractive option for decentralized water management and air conditioning enhancement in growing cities—A review. Water (Basel).

[37] Wang X, Li H, Sodoudi S (2022). The effectiveness of cool and green roofs in mitigating urban heat island and improving human thermal comfort. Build. Environ..

[38] Statistisches Bundesamt. Bodenfläche insgesamt nach Nutzungsarten in Deutschland. https://www.destatis.de/DE/Themen/Branchen-Unternehmen/Landwirtschaft-Forstwirtschaft-Fischerei/Flaechennutzung/Tabellen/bodenflaeche-insgesamt.html (2022).

[39] World Bank. Germany: Urbanization from 2011 to 2021 [Graph]. In Statista. https://www.statista.com/statistics/455825/urbanization-in-germany/ (2022).

[40] Venter O (2016). Sixteen years of change in the global terrestrial human footprint and implications for biodiversity conservation. Nat. Commun..

[41] Auffret AG (2011). Can seed dispersal by human activity play a useful role for the conservation of European grasslands?. Appl. Veg. Sci..

[42] Hodkinson DJ, Thompson K (1997). Plant dispersal: The role of man. J. Appl. Ecol..

[43] Bullock JM (2018). Human-mediated dispersal and the rewiring of spatial networks. Trends Ecol. Evol..

[44] Bullock JM, Pufal G (2020). Human-mediated dispersal as a driver of vegetation dynamics: A conceptual synthesis. J. Veg. Sci..

[45] Staude IR (2023). Prioritize grassland restoration to bend the curve of biodiversity loss. Restor. Ecol..

[46] Hari V, Rakovec O, Markonis Y, Hanel M, Kumar R (2020). Increased future occurrences of the exceptional 2018–2019 Central European drought under global warming. Sci. Rep..

[47] Dewaelheyns V, Elsen A, Vandendriessche H, Gulinck H (2013). Garden management and soil fertility in Flemish domestic gardens. Landsc. Urban Plan..

[48] Staude IR (2021). Directional turnover towards larger-ranged plants over time and across habitats. Ecol. Lett..

[49] Frankie G (2019). Native and non-native plants attract diverse bees to urban gardens in California. J. Pollinat. Ecol..

[50] Tew NE, Baldock KCR, Vaughan IP, Bird S, Memmott J (2022). Turnover in floral composition explains species diversity and temporal stability in the nectar supply of urban residential gardens. J. Appl. Ecol..

[51] Reichard SH, White P (2001). Horticulture as a pathway of invasive plant introductions in the United States: Most invasive plants have been introduced for horticultural use by nurseries, botanical gardens, and individuals. Bioscience.

[52] Mumaw L (2017). Transforming urban gardeners into land stewards. J. Environ. Psychol..

