# Supplementary material for: Putting conservation gardening into practice
Source: Sci Rep. 2023 Aug 31;13:12671. doi: 10.1038/s41598-023-39432-8 (PMC10471578; doi:10.1038/s41598-023-39432-8)
Supplement: Supplementary file 1 — Supplementary Information. [file 41598_2023_39432_MOESM1_ESM.docx]

**Supplementary Material for**

**Putting conservation gardening into practice**

Marius Munschek^1^, Reinhard Witt^2^, Katrin Kaltofen^2^, Josiane Segar^3^, Christian Wirth^1,3,4^, Alexandra Weigelt^1,3^, Rolf A. Engelmann^1,3^ & Ingmar R. Staude^1,3^

1. *Institute of Biology, Leipzig University, Leipzig, Germany*
2. *Die Naturgartenplaner, Regensburg, Germany*
3. *Botanical Garden of the University of Leipzig, Leipzig, Germany*
4. *German Centre for Integrative Biodiversity Research (iDiv) Halle-Jena Leipzig, Leipzig, Germany*

**Content**

**Supplementary Figures:**

**S Fig 1:** Workflow for building the database underlying the R Shiny app for Conservation Gardening.

**S Fig 2:** An R Shiny application for Conservation Gardening.

**Supplementary Tables**

**Table S1:** Bibliography of German federal state Red Lists.

**Table S2:** Additional Conservation Gardening species for the balcony recommended by practitioners.

**
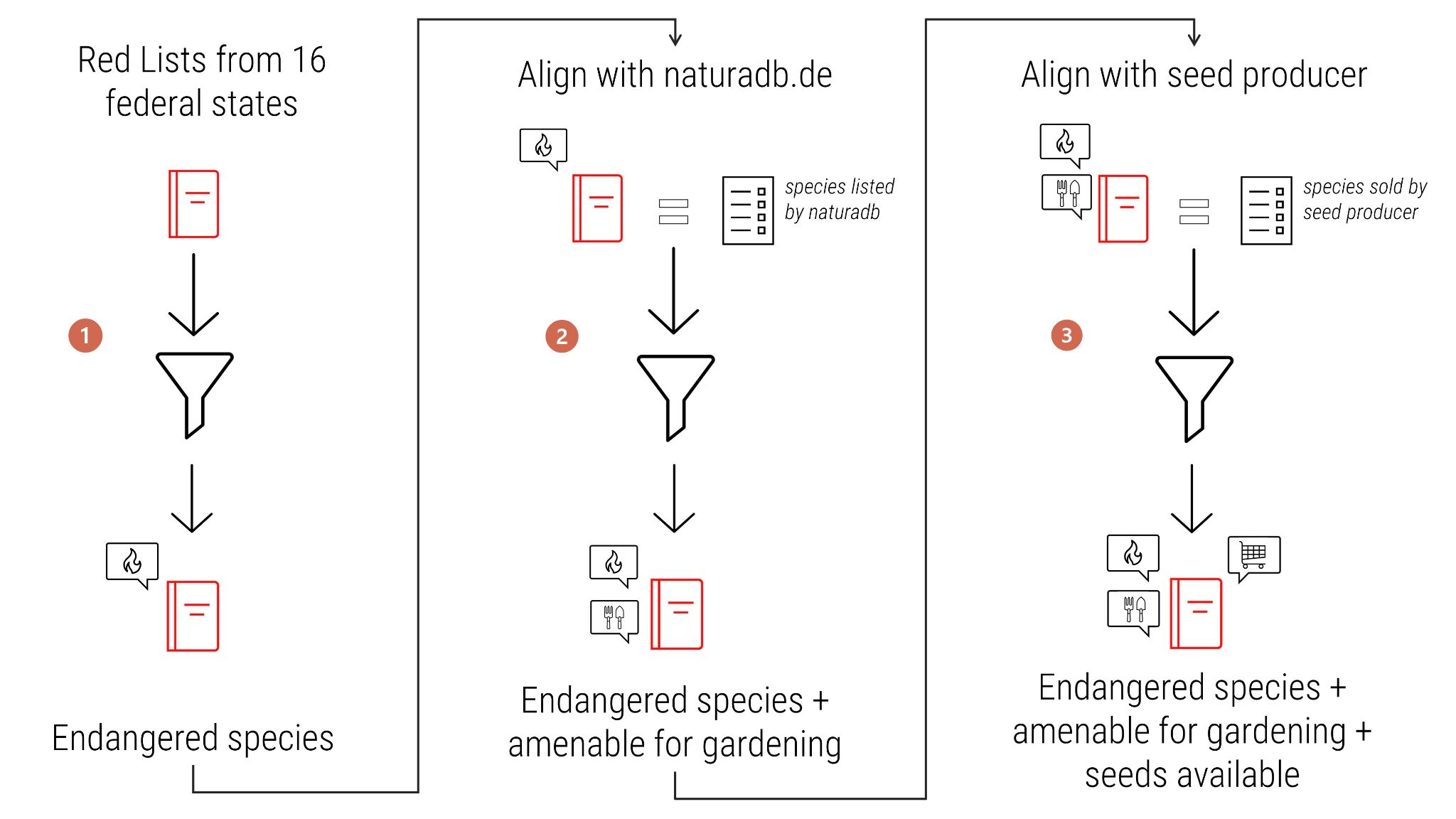
**

S Fig 1: **Workflow for building the database underlying the R Shiny app for Conservation Gardening.** 1) From each federal state Red List, we extracted and synthesized red-listed species in the following categories: 0 (Extinct or Lost), 1 (Critically Endangered), 2 (Endangered), 3 (Vulnerable), G (Endangered - Unknown Extent), R (rare), V (Near Threatened). 2) For each species on this list, we searched for entries on NaturaDB. Matching entries were kept and characteristics relevant to gardening were text mined from the website (see main text). 3) Species from step 2, i.e., species amenable to CG, were used to search the product assortments of the following seed producers: Gärtnerei Strickler, Hof Berg-Garten, Rieger-Hofmann, Gärtnerei StaudenSpatz, Blauetikett Bornträger. Matching species entries were kept and the web shop URLs of those species were queried.


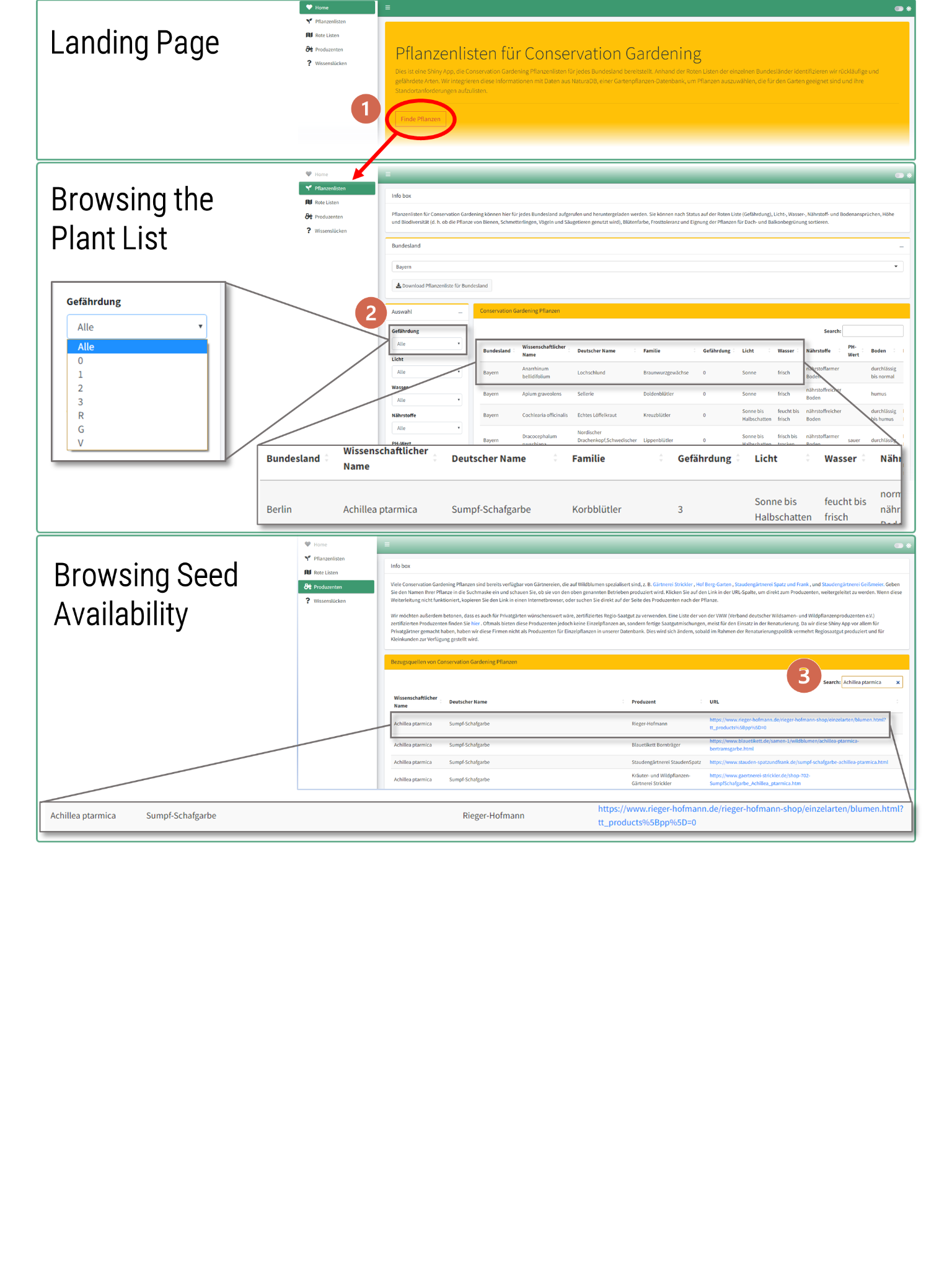


S Fig 2: **An R Shiny application for Conservation Gardening.** Screenshots provide an overview of the most important features of the app ([https://conservation-gardening.shinyapps.io/app-en/](https://conservation-gardening.shinyapps.io/application/)). The app assist users in finding plants that are amenable for CG: 1) On the Landing Page there is a short explanation of the app and a button “Find Plants”. By clicking that button the user goes to 2) the “Plant List” tab. Here the user can browse the Plant List per federal state, and filter by various criteria that can be selected in drop down menus (e.g. Red List category = 1). When the user decides for a plant, the species name can be copied into 3) the search bar under the “Producer” Tab. If the species is sold by a producer, a corresponding link to the web shop of one or producers will appear.

**Table S1: Bibliography of German federal state Red Lists.**

| Federal state | Year of publication | Original title |
| --- | --- | --- |
| *Baden-Württemberg* | 1999 | Breunig, T. & Demuth, S. (1999): Rote Liste der Farn- und Samenpflanzen Baden-Württemberg. Naturschutz-Praxis, Artenschutz 2. |
| *Bayern* | 2003 | Scheurer, M. & Ahlmer, W. (2003): Rote Liste gefährdeter Gefäßpflanzen Bayerns mit regionalisierter Florenliste. In: Schriftenreihe des Bayerischen Landesamtes für Umweltschutz. Bd. 165, Augsburg, S. 1–372. |
| *Berlin* | 2018 | Seitz, B., Ristow, M., Meißner, J., Machatzi, B., & Sukopp, H. (2018): Rote Liste und Gesamtartenliste der etablierten Farn-und Blütenpflanzen von Berlin. Universitätsverlag der TU Berlin. |
| *Brandenburg* | 2006 | Ristow, M., Herrmann, A., Illig, H., Klemm, G., Kummer, V., Kläge, H. C., ... & Zimmermann, F. (2006): Liste und Rote Liste der etablierten Gefäßpflanzen Brandenburgs. |
| *Hamburg* | 2010 | Poppendieck, H.H., Bertram, H., Brandt, I., Kreft, K.A., Kurz, H., Onnasch, A., … & Wiedemann, D. (2010):  Rote Liste und Florenliste der Gefäßpflanzen von Hamburg; Sonderdruck aus: Poppendieck, H.-H., et al. (Hrsg.): Der Hamburger Pflanzenatlas von a bis z. |
| *Hessen* | 2019 | Arbeitsgruppe „Rote Liste der  Farn- und Samenpflanzen Hessens“ der Botanischen  Vereinigung für Naturschutz in Hessen e. V. (BVNH) (2019):  Rote Liste der Farn- und Samenpflanzen Hessens – 5. Fassung |
| *Mecklenburg-Vorpommern* | 2005 | Voigtländer, U., & Henker, H. (2005): Rote Liste der Farn-und Blütenpflanzen Mecklenburg-Vorpommerns. 5. Fassung. *Schwerin: Umweltministerium Mecklenburg-Vorpommern*. |
| *Niedersachsen & Bremen* | 2004 | Garve, E. (2004): Rote Liste und Florenliste der Farn-und Blütenpflanzen in Niedersachsen und Bremen. Niedersachsen: Niedersächsisches Landesamt für Ökologie. |
| *Nordrhein-Westfalen* | 2021 | Verbücheln, G., Götte, R., Hövelmann, T., Itjeshorst, W., Keil, P., Kulbrock, P., Kulbrock, G.; Luwe, M., Mause, R., Neikes, N., Schubert, W., Schumacher, W.; Schwartze, P., van de Weyer, K. (2021): Rote Liste der Farn- und Blütenpflanzen – Pteridophyta et Spermatophyta – in Nordrhein-Westfalen. 5. Fassung, Stand Oktober 2020. LANUV-Fachbericht 118, Recklinghausen |
| *Rheinland-Pfalz* | 1988 | Ministerium für Umwelt und Gesundheit Rheinland-Pfalz (Hrsg.) Korneck, D., Lang, W. & Reichert, H. (1988): Rote Liste der in Rheinland-Pfalz ausgestorbenen, verschollenen und gefährdeten Farn- und Blütenpflanzen, Stand: 1985, 3. Auflage. Sommer; Grünstadt. |
| *Saarland* | 2020 | Schneider, T., Caspari, S., Schneider, C., & Weicherding, F. J. (2020). Rote Liste und Gesamtartenliste der Gefäßpflanzen (Tracheophyta) des Saarlandes. |
| *Sachsen* | 2013 | Schulz, D. (2013). Rote Liste und Artenliste Sachsens-Farn-und Samenpflanzen. |
| *Sachsen-Anhalt* | 2020 | Frank, D., Brade, P., Elias, D., Glowka, B., Hoch, A., John, H., ... & Wegener, U. (2020). Rote Listen Sachsen-Anhalt. Farne und Blütenpflanzen (Pteridophyta et Spermatophyta)(4. Fassung, Stand: September 2019). *Ber. Landesamt. Umweltschutz Sachsen-Anh.(Halle)*, *1*(2020), 151-186. |
| *Schleswig-Holstein* | 2021 | Romahn, K. (2021): Die Farn- und Blütenpflanzen Schleswig-Holsteins – Rote Liste, 2 Bände.– Unter Mitarbeit von Hebbel, J., Christensen, E., Kieckbusch, J., Breuer, J., Behrends, Th., Borcherding, R., Dolnik, C., Gehrken, K., Gettner, S., Haacks, M., Hamann, U., Horst, E., Jansen, W., Jödicke, K., Kellner, S., Kresken, G.-U., Lütt, S., Piontkowski, H.-U., Ruhmann, U., Stuhr, J., Timmermann-Trosiener, I., Triebstein, C. & Voss, K. Landesamt für Landwirtschaft, Umwelt und ländliche Räume des Landes Schleswig-Holstein, Flintbek (Hrsg.). |
| *Thüringen* | 2010 | Korsch, H. & W. Westhus (2010): Rote Liste der Farn- und Blütenpflanzen (Pteridophyta et Spermato- phyta) Thüringens, 5. Fassung, Stand: 10/2010 |

**Table S2: Additional Conservation Gardening species for the balcony recommended by practitioners.**

| Federal state | Species | Red List |
| --- | --- | --- |
| ***Baden-Württemberg*** | *Achillea nobilis* | 3 |
|  | *Allium lusitanicum/senescens* | 3 |
|  | *Allium sphaerocephalon* | 3 |
|  | *Allium suaveolens* | 3 |
|  | *Anchusa officinalis* | 3 |
|  | *Anemone sylvestris* | 2 |
|  | *Antennaria dioica* | 2 |
|  | *Anthemis tinctoria* | 3 |
|  | *Anthericum liliago* | 3 |
|  | *Armeria maritima* | 1 |
|  | *Artemisia pontica* | 1 |
|  | *Asperula tinctoria* | 2 |
|  | *Aster linosyris* | 3 |
|  | *Campanula cochleariifolia* | 3 |
|  | *Centaurea pseudophrygia* | 3 |
|  | *Crocus vernus ssp. albiflorus* | 1 |
|  | *Dianthus deltoides* | 3 |
|  | *Dianthus gratianopolitanus* | 3 |
|  | *Dianthus superbus* | 3 |
|  | *Dictamnus albus* | 3 |
|  | *Euphorbia seguieriana* | 2 |
|  | *Fritillaria meleagris* | 1 |
|  | *Globularia bisnagarica* | 3 |
|  | *Gypsophila repens* | 0 |
|  | *Hyssopus officinalis* | R |
|  | *Inula hirta* | 3 |
|  | *Linum flavum* | 2 |
|  | *Muscari botryoides* | 3 |
|  | *Muscari comosum* | 3 |
|  | *Muscari neglectum* | 3 |
|  | *Ononis natrix* | 0 |
|  | *Potentilla alba* | 2 |
|  | *Potentilla rupestris* | 2 |
|  | *Primula auricula* | 3 |
|  | *Primula vulgaris* | 2 |
|  | *Pulsatilla vulgaris* | 3 |
|  | *Salvia nemorosa* | 2 |
|  | *Scabiosa canescens* | 2 |
|  | *Silene viscaria* | 3 |
|  | *Teucrium montanum* | 3 |
|  | *Thymus serpyllum* | 2 |
|  | *Trifolium montanum* | 3 |
|  | *Trifolium rubens* | 3 |
|  | *Tulipa sylvestris* | 3 |
| ***Bayern*** | *Achillea nobilis* | 2 |
|  | *Allium sphaerocephalon* | 3 |
|  | *Alyssum montanum* | 2 |
|  | *Antennaria dioica* | 3 |
|  | *Anthericum liliago* | 3 |
|  | *Armeria maritima* | 3 |
|  | *Asperula tinctoria* | 3 |
|  | *Aster amellus* | 3 |
|  | *Aster linosyris* | 3 |
|  | *Calamintha menthifolia* | 2 |
|  | *Calamintha nepeta* | 1 |
|  | *Centaurea nigra* | 3 |
|  | *Centaurea pseudophrygia* | 3 |
|  | *Centaurea triumfettii* | 1 |
|  | *Corydalis solida* | 3 |
|  | *Crocus vernus ssp. albiflorus* | 2 |
|  | *Dianthus gratianopolitanus* | 2 |
|  | *Dianthus superbus* | 3 |
|  | *Dianthus sylvestris* | 2 |
|  | *Dictamnus albus* | 3 |
|  | *Digitalis grandiflora* | 3 |
|  | *Dorycnium germanicum* | 3 |
|  | *Dracocephalum ruyschiana* | 0 |
|  | *Epilobium dodonaei* | 0 |
|  | *Euphorbia seguieriana* | 2 |
|  | *Filipendula vulgaris* | 3 |
|  | *Fritillaria meleagris* | 2 |
|  | *Geranium phaeum* | 2 |
|  | *Globularia bisnagarica* | 3 |
|  | *Helianthemum apenninum* | 3 |
|  | *Helianthemum nummularium* | 3 |
|  | *Helleborus foetidus* | 3 |
|  | *Inula ensifolia* | 0 |
|  | *Malva moschata* | 3 |
|  | *Melittis melissophyllum* | 3 |
|  | *Muscari botryoides* | 3 |
|  | *Muscari comosum* | 2 |
|  | *Muscari neglectum* | 2 |
|  | *Onobrychis arenaria* | 2 |
|  | *Ornithogalum vulgare/umbellatum* | 3 |
|  | *Petrorhagia saxifraga* | 3 |
|  | *Potentilla alba* | 3 |
|  | *Potentilla crantzii* | R |
|  | *Potentilla rupestris* | 1 |
|  | *Primula vulgaris* | 2 |
|  | *Salvia nemorosa* | 2 |
|  | *Scabiosa canescens* | 2 |
|  | *Scilla bifolia* | 3 |
|  | *Silene viscaria* | 3 |
|  | *Thymus serpyllum* | 3 |
|  | *Trifolium rubens* | 3 |
| ***Berlin*** | *Ajuga genevensis* | 3 |
|  | *Ajuga reptans* | 1 |
|  | *Allium lusitanicum/senescens* | 0 |
|  | *Antennaria dioica* | 0 |
|  | *Anthemis tinctoria* | G |
|  | *Anthericum liliago* | 2 |
|  | *Anthericum ramosum* | 2 |
|  | *Anthyllis vulneraria* | 1 |
|  | *Asperula cynanchica* | 0 |
|  | *Asperula tinctoria* | 1 |
|  | *Betonica officinalis* | 2 |
|  | *Campanula glomerata* | 0 |
|  | *Campanula persicifolia* | 2 |
|  | *Campanula trachelium* | 0 |
|  | *Dianthus carthusianorum* | 1 |
|  | *Dianthus deltoides* | 3 |
|  | *Dianthus superbus* | 1 |
|  | *Filipendula vulgaris* | 2 |
|  | *Geranium sanguineum* | 1 |
|  | *Helianthemum nummularium* | 1 |
|  | *Helleborus foetidus* | R |
|  | *Hepatica nobilis* | 1 |
|  | *Inula salicina* | 1 |
|  | *Lathyrus tuberosus* | 3 |
|  | *Malva alcea* | 3 |
|  | *Malva moschata* | 3 |
|  | *Ononis spinosa* | 0 |
|  | *Pimpinella saxifraga* | 3 |
|  | *Potentilla alba* | 1 |
|  | *Primula veris* | 1 |
|  | *Prunella grandiflora* | 0 |
|  | *Pulsatilla vulgaris* | 0 |
|  | *Salvia pratensis* | G |
|  | *Scabiosa canescens* | 1 |
|  | *Scabiosa columbaria* | 1 |
|  | *Silene nutans* | 2 |
|  | *Stachys recta* | 0 |
|  | *Thymus serpyllum* | 2 |
|  | *Trifolium montanum* | 0 |
|  | *Veronica spicata* | 1 |
|  | *Veronica teucrium* | 0 |
| ***Brandenburg*** | *Allium lusitanicum/senescens* | 1 |
|  | *Alyssum montanum* | 1 |
|  | *Anemone sylvestris* | 2 |
|  | *Antennaria dioica* | 1 |
|  | *Anthericum liliago* | 3 |
|  | *Anthericum ramosum* | 3 |
|  | *Anthyllis vulneraria* | 3 |
|  | *Asperula cynanchica* | 2 |
|  | *Asperula tinctoria* | 3 |
|  | *Aster amellus* | 1 |
|  | *Aster linosyris* | 3 |
|  | *Astrantia major* | 1 |
|  | *Betonica officinalis* | 2 |
|  | *Campanula glomerata* | 2 |
|  | *Campanula sibirica* | 3 |
|  | *Dianthus arenarius* | 1 |
|  | *Dianthus carthusianorum* | 3 |
|  | *Dianthus deltoides* | 3 |
|  | *Dianthus gratianopolitanus* | 1 |
|  | *Dianthus superbus* | 2 |
|  | *Digitalis grandiflora* | 2 |
|  | *Filipendula vulgaris* | 2 |
|  | *Fritillaria meleagris* | 1 |
|  | *Geranium sanguineum* | 2 |
|  | *Helianthemum nummularium* | G |
|  | *Helleborus foetidus* | R |
|  | *Inula hirta* | 1 |
|  | *Inula salicina* | 2 |
|  | *Lathyrus niger* | 2 |
|  | *Malva moschata* | 3 |
|  | *Melittis melissophyllum* | 1 |
|  | *Muscari comosum* | 1 |
|  | *Onobrychis viciifolia* | 3 |
|  | *Ononis spinosa* | 3 |
|  | *Origanum vulgare* | 3 |
|  | *Potentilla alba* | 2 |
|  | *Potentilla rupestris* | 0 |
|  | *Potentilla verna* | 3 |
|  | *Primula elatior* | 1 |
|  | *Primula veris* | 3 |
|  | *Prunella grandiflora* | 2 |
|  | *Pulsatilla vulgaris* | 1 |
|  | *Salvia pratensis* | 3 |
|  | *Salvia verticillata* | 3 |
|  | *Sanguisorba minor* | 3 |
|  | *Scabiosa canescens* | 2 |
|  | *Scabiosa columbaria* | 2 |
|  | *Scabiosa ochroleuca* | 2 |
|  | *Silene viscaria* | 2 |
|  | *Stachys recta* | 3 |
|  | *Thymus pannonicus* | 1 |
|  | *Trifolium rubens* | 1 |
|  | *Tulipa sylvestris* | 2 |
|  | *Veronica spicata* | 3 |
|  | *Veronica teucrium* | 2 |
| ***Bremen/Niedersachsen*** | *Ajuga genevensis* | 0 |
|  | *Allium lusitanicum/senescens* | 1 |
|  | *Anemone sylvestris* | 2 |
|  | *Antennaria dioica* | 2 |
|  | *Anthericum liliago* | 2 |
|  | *Anthericum ramosum* | 1 |
|  | *Aquilegia vulgaris* | 3 |
|  | *Asperula cynanchica* | 2 |
|  | *Asperula tinctoria* | 0 |
|  | *Aster amellus* | 1 |
|  | *Aster linosyris* | 1 |
|  | *Buglossoides purpurocaerulea* | 3 |
|  | *Campanula glomerata* | 2 |
|  | *Centaurea montana* | 2 |
|  | *Dianthus carthusianorum* | 3 |
|  | *Dianthus deltoides* | 3 |
|  | *Dianthus gratianopolitanus* | R |
|  | *Dianthus superbus* | 1 |
|  | *Dictamnus albus* | 0 |
|  | *Digitalis grandiflora* | 2 |
|  | *Euphorbia seguieriana* | 0 |
|  | *Filipendula vulgaris* | 2 |
|  | *Fritillaria meleagris* | 3 |
|  | *Geranium sanguineum* | 2 |
|  | *Gypsophila repens* | R |
|  | *Helianthemum nummularium* | 3 |
|  | *Hippocrepis comosa* | 3 |
|  | *Inula hirta* | R |
|  | *Inula salicina* | 3 |
|  | *Lathyrus niger* | 2 |
|  | *Melittis melissophyllum* | 1 |
|  | *Polygonatum odoratum* | 2 |
|  | *Potentilla alba* | 1 |
|  | *Primula vulgaris* | 1 |
|  | *Prunella grandiflora* | 3 |
|  | *Salvia pratensis* | 3 |
|  | *Saxifraga granulata* | 3 |
|  | *Scabiosa canescens* | 2 |
|  | *Stachys recta* | 2 |
|  | *Tanacetum corymbosum* | 3 |
|  | *Thymus praecox* | R |
|  | *Thymus serpyllum* | 3 |
|  | *Trifolium montanum* | 0 |
|  | *Trifolium rubens* | 3 |
|  | *Veronica spicata* | 2 |
| ***Hamburg*** | *Agrimonia eupatoria* | 3 |
|  | *Agrimonia procera* | 2 |
|  | *Ajuga genevensis* | 0 |
|  | *Allium schoenoprasum* | 3 |
|  | *Antennaria dioica* | 0 |
|  | *Anthericum liliago* | 0 |
|  | *Anthericum ramosum* | 1 |
|  | *Anthyllis vulneraria* | 2 |
|  | *Armeria maritima* | 1 |
|  | *Buglossoides purpurocaerulea* | R |
|  | *Campanula persicifolia* | 0 |
|  | *Campanula rotundifolia* | 3 |
|  | *Campanula trachelium* | 1 |
|  | *Centaurea jacea* | 3 |
|  | *Centaurea nigra* | R |
|  | *Centaurea pseudophrygia* | 2 |
|  | *Centaurea scabiosa* | 1 |
|  | *Dianthus carthusianorum* | 1 |
|  | *Dianthus deltoides* | 1 |
|  | *Echium vulgare* | 3 |
|  | *Filipendula vulgaris* | 0 |
|  | *Fritillaria meleagris* | 1 |
|  | *Galium verum* | 3 |
|  | *Hepatica nobilis* | 0 |
|  | *Hieracium murorum* | 2 |
|  | *Hieracium umbellatum* | 2 |
|  | *Ononis spinosa* | 1 |
|  | *Pimpinella saxifraga* | 1 |
|  | *Polygonatum odoratum* | 0 |
|  | *Primula elatior* | 2 |
|  | *Pulsatilla vulgaris* | 0 |
|  | *Saxifraga granulata* | 1 |
|  | *Scabiosa columbaria* | 1 |
|  | *Sedum maximum* | 3 |
|  | *Sedum telephium* | 3 |
|  | *Silene nutans* | 0 |
|  | *Silene vulgaris* | 3 |
|  | *Solidago virgaurea* | 3 |
|  | *Succisa pratensis* | 1 |
|  | *Thymus pulegioides* | 2 |
|  | *Thymus serpyllum* | 1 |
|  | *Veronica spicata* | 0 |
| ***Hessen*** | *Achillea nobilis* | 3 |
|  | *Allium sphaerocephalon* | 2 |
|  | *Alyssum montanum* | R |
|  | *Anemone sylvestris* | 2 |
|  | *Antennaria dioica* | 2 |
|  | *Anthericum liliago* | 3 |
|  | *Anthericum ramosum* | 3 |
|  | *Armeria maritima* | 3 |
|  | *Asperula tinctoria* | 0 |
|  | *Aster amellus* | 2 |
|  | *Aster linosyris* | 3 |
|  | *Calamintha menthifolia* | 3 |
|  | *Dianthus gratianopolitanus* | 3 |
|  | *Dianthus superbus* | 2 |
|  | *Dictamnus albus* | 2 |
|  | *Euphorbia seguieriana* | 2 |
|  | *Filipendula vulgaris* | 2 |
|  | *Globularia bisnagarica* | 0 |
|  | *Inula hirta* | 1 |
|  | *Inula salicina* | 3 |
|  | *Lathyrus niger* | 3 |
|  | *Melittis melissophyllum* | 0 |
|  | *Muscari comosum* | 3 |
|  | *Muscari neglectum* | 3 |
|  | *Onobrychis arenaria* | 0 |
|  | *Potentilla alba* | 2 |
|  | *Pulsatilla vulgaris* | 3 |
|  | *Scabiosa canescens* | 3 |
|  | *Scabiosa ochroleuca* | 0 |
|  | *Teucrium chamaedrys* | 3 |
|  | *Teucrium montanum* | R |
|  | *Thymus serpyllum* | 3 |
|  | *Trifolium rubens* | 1 |
|  | *Veronica spicata* | 2 |
| ***Mecklenburg-Vorpommern*** | *Ajuga genevensis* | 3 |
|  | *Allium lusitanicum/senescens* | 1 |
|  | *Allium schoenoprasum* | R |
|  | *Anemone sylvestris* | 0 |
|  | *Antennaria dioica* | 1 |
|  | *Anthericum liliago* | 1 |
|  | *Anthericum ramosum* | 1 |
|  | *Armeria maritima* | 3 |
|  | *Asperula cynanchica* | 1 |
|  | *Asperula tinctoria* | 0 |
|  | *Aster linosyris* | 1 |
|  | *Betonica officinalis* | 2 |
|  | *Campanula glomerata* | 2 |
|  | *Campanula sibirica* | 1 |
|  | *Centaurea jacea* | 3 |
|  | *Centaurea nigra* | 1 |
|  | *Centaurea pseudophrygia* | 1 |
|  | *Corydalis solida* | R |
|  | *Dianthus arenarius* | 1 |
|  | *Dianthus carthusianorum* | 3 |
|  | *Dianthus deltoides* | 3 |
|  | *Dianthus superbus* | 2 |
|  | *Digitalis grandiflora* | 1 |
|  | *Geranium sanguineum* | 1 |
|  | *Helianthemum nummularium* | 2 |
|  | *Helleborus foetidus* | R |
|  | *Inula hirta* | 1 |
|  | *Inula salicina* | 2 |
|  | *Lathyrus tuberosus* | 2 |
|  | *Limonium vulgare* | 2 |
|  | *Onobrychis viciifolia* | 2 |
|  | *Ononis spinosa* | 3 |
|  | *Potentilla alba* | 1 |
|  | *Potentilla recta* | 2 |
|  | *Primula vulgaris* | 0 |
|  | *Prunella grandiflora* | 1 |
|  | *Pulsatilla vulgaris* | 1 |
|  | *Salvia pratensis* | 3 |
|  | *Salvia verticillata* | 1 |
|  | *Saxifraga granulata* | 1 |
|  | *Scabiosa canescens* | 0 |
|  | *Scabiosa columbaria* | 3 |
|  | *Sedum telephium* | 1 |
|  | *Stachys recta* | 2 |
|  | *Succisa pratensis* | 2 |
|  | *Tanacetum corymbosum* | 0 |
|  | *Thymus serpyllum* | 3 |
|  | *Trifolium montanum* | 2 |
|  | *Trifolium rubens* | 0 |
|  | *Tulipa sylvestris* | R |
|  | *Veronica spicata* | 3 |
|  | *Veronica teucrium* | 2 |
| ***Nordrhein-Westfalen*** | *Achillea nobilis* | 0 |
|  | *Ajuga genevensis* | 3 |
|  | *Allium sphaerocephalon* | 0 |
|  | *Alyssum montanum* | 1 |
|  | *Anemone sylvestris* | 1 |
|  | *Anthemis tinctoria* | 3 |
|  | *Anthericum liliago* | 3 |
|  | *Anthericum ramosum* | R |
|  | *Anthyllis vulneraria* | 3 |
|  | *Armeria maritima* | 3 |
|  | *Artemisia pontica* | 1 |
|  | *Asperula cynanchica* | 3 |
|  | *Aster amellus* | 1 |
|  | *Betonica officinalis* | 3 |
|  | *Calamintha menthifolia* | 3 |
|  | *Campanula glomerata* | 3 |
|  | *Centaurea pseudophrygia* | 2 |
|  | *Dianthus carthusianorum* | 3 |
|  | *Dianthus deltoides* | 3 |
|  | *Dianthus superbus* | 3 |
|  | *Dictamnus albus* | 0 |
|  | *Digitalis grandiflora* | 2 |
|  | *Digitalis lutea* | 3 |
|  | *Euphorbia seguieriana* | 1 |
|  | *Filipendula vulgaris* | 2 |
|  | *Fritillaria meleagris* | 1 |
|  | *Geranium sanguineum* | 3 |
|  | *Helianthemum nummularium* | 3 |
|  | *Helleborus foetidus* | R |
|  | *Hepatica nobilis* | 3 |
|  | *Hieracium umbellatum* | 3 |
|  | *Hyssopus officinalis* | 0 |
|  | *Lathyrus niger* | 3 |
|  | *Malva alcea* | 3 |
|  | *Muscari botryoides* | 2 |
|  | *Muscari neglectum* | 2 |
|  | *Ononis spinosa* | 3 |
|  | *Polygonatum odoratum* | 3 |
|  | *Primula vulgaris* | 2 |
|  | *Prunella grandiflora* | 3 |
|  | *Pulsatilla vulgaris* | 3 |
|  | *Salvia verticillata* | 3 |
|  | *Saxifraga granulata* | 3 |
|  | *Scilla bifolia* | R |
|  | *Silene nutans* | 3 |
|  | *Stachys recta* | 3 |
|  | *Succisa pratensis* | 3 |
|  | *Tanacetum corymbosum* | 2 |
|  | *Teucrium montanum* | 3 |
|  | *Thymus praecox* | 3 |
|  | *Thymus pulegioides* | 3 |
|  | *Thymus serpyllum* | 2 |
|  | *Trifolium montanum* | 3 |
|  | *Trifolium rubens* | 1 |
|  | *Tulipa sylvestris* | 2 |
|  | *Veronica spicata* | 1 |
|  | *Veronica teucrium* | 3 |
| ***Rheinland-Pfalz*** | *Allium schoenoprasum* | 3 |
|  | *Allium sphaerocephalon* | 3 |
|  | *Anemone sylvestris* | 2 |
|  | *Antennaria dioica* | 2 |
|  | *Armeria maritima* | 0 |
|  | *Asperula tinctoria* | 2 |
|  | *Aster amellus* | 3 |
|  | *Dianthus gratianopolitanus* | 3 |
|  | *Dianthus superbus* | 2 |
|  | *Euphorbia seguieriana* | 3 |
|  | *Filipendula vulgaris* | 3 |
|  | *Fritillaria meleagris* | 0 |
|  | *Globularia bisnagarica* | 2 |
|  | *Helianthemum apenninum* | 2 |
|  | *Hepatica nobilis* | 3 |
|  | *Inula hirta* | 2 |
|  | *Inula salicina* | 3 |
|  | *Melittis melissophyllum* | 2 |
|  | *Muscari comosum* | 2 |
|  | *Muscari neglectum* | 2 |
|  | *Narcissus pseudonarcissus* | 3 |
|  | *Potentilla alba* | 2 |
|  | *Potentilla rupestris* | 3 |
|  | *Pulsatilla vulgaris* | 3 |
|  | *Saxifraga paniculata* | 3 |
|  | *Scabiosa canescens* | 2 |
|  | *Teucrium montanum* | 3 |
|  | *Thymus serpyllum* | 2 |
|  | *Trifolium rubens* | 3 |
|  | *Tulipa sylvestris* | 2 |
|  | *Veronica spicata* | 3 |
| ***Saarland*** | *Anemone sylvestris* | 0 |
|  | *Antennaria dioica* | 0 |
|  | *Anthemis tinctoria* | 3 |
|  | *Anthericum liliago* | 3 |
|  | *Anthericum ramosum* | R |
|  | *Armeria maritima* | 3 |
|  | *Asperula cynanchica* | 3 |
|  | *Aster amellus* | 2 |
|  | *Campanula glomerata* | 3 |
|  | *Centaurea montana* | 0 |
|  | *Dianthus carthusianorum* | R |
|  | *Dianthus deltoides* | 3 |
|  | *Digitalis lutea* | 3 |
|  | *Erysimum cheiri* | R |
|  | *Globularia bisnagarica* | 0 |
|  | *Hippocrepis comosa* | 3 |
|  | *Lathyrus vernus* | 0 |
|  | *Muscari botryoides* | 2 |
|  | *Muscari comosum* | 1 |
|  | *Muscari neglectum* | R |
|  | *Narcissus pseudonarcissus* | 1 |
|  | *Ononis spinosa* | R |
|  | *Polygonatum odoratum* | R |
|  | *Primula veris* | 3 |
|  | *Prunella grandiflora* | R |
|  | *Pulsatilla vulgaris* | 2 |
|  | *Stachys recta* | 2 |
|  | *Tanacetum corymbosum* | 1 |
|  | *Teucrium chamaedrys* | 3 |
|  | *Teucrium montanum* | 2 |
|  | *Thymus praecox* | 3 |
|  | *Trifolium montanum* | 3 |
|  | *Trifolium rubens* | 2 |
|  | *Veronica teucrium* | 2 |
| ***Sachsen*** | *Achillea nobilis* | 1 |
|  | *Agrimonia procera* | 2 |
|  | *Ajuga genevensis* | 3 |
|  | *Allium lusitanicum/senescens* | 2 |
|  | *Alyssum montanum* | 1 |
|  | *Anemone sylvestris* | 0 |
|  | *Antennaria dioica* | 1 |
|  | *Anthemis tinctoria* | 1 |
|  | *Anthericum liliago* | 3 |
|  | *Anthericum ramosum* | 3 |
|  | *Asperula cynanchica* | 1 |
|  | *Asperula tinctoria* | 0 |
|  | *Aster amellus* | 0 |
|  | *Aster linosyris* | 0 |
|  | *Astrantia major* | 2 |
|  | *Betonica officinalis* | 3 |
|  | *Campanula glomerata* | 1 |
|  | *Centaurea pseudophrygia* | 3 |
|  | *Centaurea scabiosa* | 3 |
|  | *Corydalis solida* | 2 |
|  | *Dianthus carthusianorum* | 3 |
|  | *Dianthus gratianopolitanus* | 2 |
|  | *Digitalis grandiflora* | 3 |
|  | *Euphorbia seguieriana* | 0 |
|  | *Filipendula vulgaris* | 2 |
|  | *Geranium sanguineum* | 2 |
|  | *Helianthemum nummularium* | 2 |
|  | *Hepatica nobilis* | 2 |
|  | *Inula hirta* | 1 |
|  | *Inula salicina* | 2 |
|  | *Knautia drymeia* | R |
|  | *Lathyrus niger* | 2 |
|  | *Lathyrus tuberosus* | 3 |
|  | *Malva alcea* | 3 |
|  | *Melittis melissophyllum* | 1 |
|  | *Muscari comosum* | 1 |
|  | *Muscari neglectum* | 3 |
|  | *Ononis spinosa* | 2 |
|  | *Polygonatum odoratum* | 3 |
|  | *Potentilla alba* | 1 |
|  | *Potentilla recta* | 3 |
|  | *Potentilla rupestris* | 1 |
|  | *Primula veris* | 3 |
|  | *Prunella grandiflora* | 0 |
|  | *Pulsatilla vulgaris* | 1 |
|  | *Salvia nemorosa* | 1 |
|  | *Salvia pratensis* | 3 |
|  | *Salvia verticillata* | 3 |
|  | *Scabiosa canescens* | 1 |
|  | *Scabiosa columbaria* | 2 |
|  | *Scabiosa ochroleuca* | 3 |
|  | *Solidago virgaurea ssp. minuta* | R |
|  | *Stachys recta* | 2 |
|  | *Tanacetum corymbosum* | 1 |
|  | *Thymus pannonicus* | 0 |
|  | *Thymus praecox* | 0 |
|  | *Thymus serpyllum* | 3 |
|  | *Trifolium montanum* | 1 |
|  | *Trifolium rubens* | 0 |
|  | *Tulipa sylvestris* | 1 |
|  | *Veronica spicata* | 1 |
|  | *Veronica teucrium* | 2 |
| ***Sachsen-Anhalt*** | *Achillea nobilis* | 3 |
|  | *Allium lusitanicum/senescens* | 3 |
|  | *Allium sphaerocephalon* | 0 |
|  | *Alyssum montanum* | 3 |
|  | *Anemone sylvestris* | 3 |
|  | *Antennaria dioica* | 1 |
|  | *Anthericum liliago* | 3 |
|  | *Anthericum ramosum* | 3 |
|  | *Artemisia pontica* | 1 |
|  | *Aster alpinus* | R |
|  | *Aster amellus* | 3 |
|  | *Aster linosyris* | 3 |
|  | *Astrantia major* | 2 |
|  | *Betonica officinalis* | 3 |
|  | *Campanula glomerata* | 3 |
|  | *Centaurea pseudophrygia* | 3 |
|  | *Dianthus gratianopolitanus* | R |
|  | *Dianthus superbus* | 2 |
|  | *Dictamnus albus* | 3 |
|  | *Digitalis grandiflora* | 3 |
|  | *Dracocephalum ruyschiana* | 0 |
|  | *Euphorbia seguieriana* | 3 |
|  | *Geranium sanguineum* | 3 |
|  | *Globularia bisnagarica* | 3 |
|  | *Inula hirta* | 3 |
|  | *Iris aphylla* | 2 |
|  | *Koeleria glauca* | 2 |
|  | *Malva alcea* | 3 |
|  | *Melittis melissophyllum* | 2 |
|  | *Muscari botryoides* | 0 |
|  | *Muscari comosum* | 1 |
|  | *Onobrychis arenaria* | 2 |
|  | *Potentilla alba* | 3 |
|  | *Prunella grandiflora* | 3 |
|  | *Pulsatilla vulgaris* | 2 |
|  | *Salvia nemorosa* | 3 |
|  | *Scabiosa canescens* | 3 |
|  | *Scabiosa columbaria* | 3 |
|  | *Scilla bifolia* | 2 |
|  | *Trifolium montanum* | 3 |
|  | *Trifolium rubens* | 2 |
|  | *Veronica spicata* | 3 |
|  | *Veronica teucrium* | 3 |
| ***Schleswig-Holstein*** | *Agrimonia eupatoria* | 3 |
|  | *Agrimonia procera* | 3 |
|  | *Ajuga genevensis* | 2 |
|  | *Allium lusitanicum/senescens* | 1 |
|  | *Allium schoenoprasum* | 0 |
|  | *Antennaria dioica* | 1 |
|  | *Anthemis tinctoria* | 1 |
|  | *Anthericum liliago* | 1 |
|  | *Anthericum ramosum* | 1 |
|  | *Anthyllis vulneraria* | 3 |
|  | *Betonica officinalis* | 1 |
|  | *Campanula glomerata* | 1 |
|  | *Campanula persicifolia* | 1 |
|  | *Campanula rotundifolia* | 3 |
|  | *Centaurea scabiosa* | 2 |
|  | *Clinopodium vulgare* | 3 |
|  | *Dianthus carthusianorum* | 1 |
|  | *Dianthus deltoides* | 2 |
|  | *Filipendula vulgaris* | 1 |
|  | *Geranium sanguineum* | 1 |
|  | *Hepatica nobilis* | 1 |
|  | *Hieracium murorum* | 3 |
|  | *Hieracium umbellatum* | 3 |
|  | *Inula salicina* | 1 |
|  | *Koeleria glauca* | 2 |
|  | *Lathyrus niger* | 1 |
|  | *Lathyrus vernus* | 1 |
|  | *Lotus corniculatus* | 3 |
|  | *Origanum vulgare* | 2 |
|  | *Pimpinella saxifraga* | 3 |
|  | *Polygonatum odoratum* | 1 |
|  | *Potentilla recta* | 3 |
|  | *Potentilla verna* | 1 |
|  | *Primula veris* | 2 |
|  | *Primula vulgaris* | 2 |
|  | *Pulsatilla vulgaris* | 1 |
|  | *Saxifraga granulata* | 3 |
|  | *Scabiosa columbaria* | 2 |
|  | *Silene nutans* | 2 |
|  | *Thymus pulegioides* | 3 |
|  | *Thymus serpyllum* | 2 |
| ***Thüringen*** | *Allium schoenoprasum* | 3 |
|  | *Anchusa officinalis* | 3 |
|  | *Antennaria dioica* | 3 |
|  | *Artemisia pontica* | 1 |
|  | *Asperula tinctoria* | 2 |
|  | *Aster alpinus* | 2 |
|  | *Aster amellus* | 3 |
|  | *Aster linosyris* | 3 |
|  | *Astrantia major* | 2 |
|  | *Betonica officinalis* | 3 |
|  | *Calamintha menthifolia* | 1 |
|  | *Campanula glomerata* | 3 |
|  | *Centaurea montana* | 2 |
|  | *Centaurea nigra* | 2 |
|  | *Corydalis solida* | 1 |
|  | *Dianthus gratianopolitanus* | 1 |
|  | *Dianthus superbus* | 2 |
|  | *Dictamnus albus* | 3 |
|  | *Digitalis grandiflora* | 3 |
|  | *Euphorbia seguieriana* | 2 |
|  | *Filipendula vulgaris* | 3 |
|  | *Globularia bisnagarica* | 3 |
|  | *Helianthemum nummularium* | 2 |
|  | *Inula hirta* | 3 |
|  | *Melittis melissophyllum* | 2 |
|  | *Muscari botryoides* | 2 |
|  | *Muscari comosum* | 2 |
|  | *Potentilla alba* | 2 |
|  | *Potentilla recta* | 1 |
|  | *Potentilla rupestris* | 3 |
|  | *Prunella grandiflora* | 1 |
|  | *Scilla bifolia* | 3 |
|  | *Sedum telephium* | 3 |
|  | *Teucrium montanum* | 2 |
|  | *Thymus serpyllum* | 0 |
|  | *Trifolium rubens* | 3 |
|  | *Tulipa sylvestris* | 1 |
